# Theoretical Study of Aluminum Hydroxide as a Hydrogen-Bonded Layered Material

**DOI:** 10.3390/nano8060375

**Published:** 2018-05-28

**Authors:** Dongwook Kim, Jong Hyun Jung, Jisoon Ihm

**Affiliations:** 1 Department of Physics and Astronomy, Seoul National University, Seoul 08826, Korea; dwwwkim@gmail.com (D.K.); jjhjm17@snu.ac.kr (J.H.J.); 2 Department of Physics, Pohang University of Science and Technology, Pohang 37673, Korea

**Keywords:** aluminum hydroxide, Bayerite, Gibbsite, two-dimensional layered material, hydrogen bonding, density functional theory, first-principles calculation, band structure, band gap, surface states, alkali-halide intercalation

## Abstract

In many layer-structured materials, constituent layers are bound through van der Waals (vdW) interactions. However, hydrogen bonding is another type of weak interaction which can contribute to the formation of multi-layered materials. In this work, we investigate aluminum hydroxide [Al(OH)${}_{3}$] having hydrogen bonding as an interlayer binding mechanism. We study the crystal structures and electronic band structures of bulk, single-layer, and multi-layer Al(OH)${}_{3}$ using density functional theory calculations. We find that hydrogen bonds across the constituent layers indeed give rise to interlayer binding stronger than vdW interactions, and a reduction of the band gap occurs for an isolated layer as compared to bulk Al(OH)${}_{3}$ which is attributed to the emergence of surface states. We also consider the alkali-halide intercalation between layers and examine how the intercalated atoms affect the atomic and electronic structures of Al(OH)${}_{3}$.

## 1. Introduction

Since the successful exfoliation of a single layer graphene from graphite [1], there has been ongoing interest in two-dimensional (2D) (single-layered or multi-layered) materials. Research on graphene [1,2,3,4,5], hexagonal boron nitride (h-BN) [6,7,8,9,10] and various transition metal dichalcogenides (TMDs) [11,12,13] have been reported to mention a few. In the case of multi-layered materials, the stacking sequence and the number of stacked layers have diverse effects on their physical properties. For example, there is a transition of the band gap from direct to indirect in MoS${}_{2}$ depending on the number of layers [14]. The kinds of atoms constituting heterostructures or multi-layered materials also affect the electronic band structures and optical properties. In addition to fundamental research, 2D single-layered or multi-layered materials have been studied due to their potential applications, for example, in energy storage [15,16], gas adsorption, catalysis [17], and energy conversion [16]. Most of the above-mentioned and recently reported layered materials have a common feature that they have the van der Waals (vdW) interaction as an interlayer binding mechanism. On the other hand, there is another well-known interaction for weak interlayer binding, namely, the hydrogen bonding. Al(OH)${}_{3}$ with interlayer hydrogen bonds is an example which has been studied both experimentally and theoretically before [18,19,20,21,22]. However, their properties as a quasi 2D layered material have not been sufficiently appreciated compared to the vdW-interaction-mediated materials. In the present study, we take Al(OH)${}_{3}$ as a representative hydrogen-bonded layered material and show the details of the single layer structure as a building block of the bulk structure. We will show electronic band structures of the single layer, the few-layer, and the bulk Al(OH)${}_{3}$ (including delicate difference between two bulk phases, Gibbsite and Bayerite) using the density functional theory (DFT) calculations. We will also present the characteristic surface states originated from breaking interlayer hydrogen bonds. In the final section, we will report the result of the alkali-halide intercalation between layers of Al(OH)${}_{3}$.

## 2. Calculation Methods

We performed first-principles DFT electronic structure calculations using the generalized-gradient approximation (GGA) of the Perdew-Burke-Ernzerhof (PBE) exchange-correlation functional [23] implemented in the VASP code [24]. Pseudopotentials were generated through the projector augmented-wave (PAW) method [25]. The kinetic energy cutoff was set to 500 eV for electronic band calculations and 800 eV for the relaxation of unit cells. 9 × 9 × 4 Monkhorst-Pack k-point grids were used for bulk and 9 × 9 × 1 for layer calculations. In the single-layer and multi-layer calculations, we included the vacuum region thicker than 15 Å to eliminate fictitious interactions between layers across the unit supercell. To describe the interaction between layers properly, the vdW-interaction is included through the D2 method by Grimme (DFT-D2) [26]. DFT-D2 describes this type of interactions with additive pairwise correction terms which depend on dispersion coefficients ($C_{ij}$) for the atom pairs and damping functions $f(r_{ij})$ with cutoff radius $R_{0ij}$.

## 3. Results and Discussion

### 3.1. Crystal Structure of Aluminum Hydroxide

**Figure 1:**
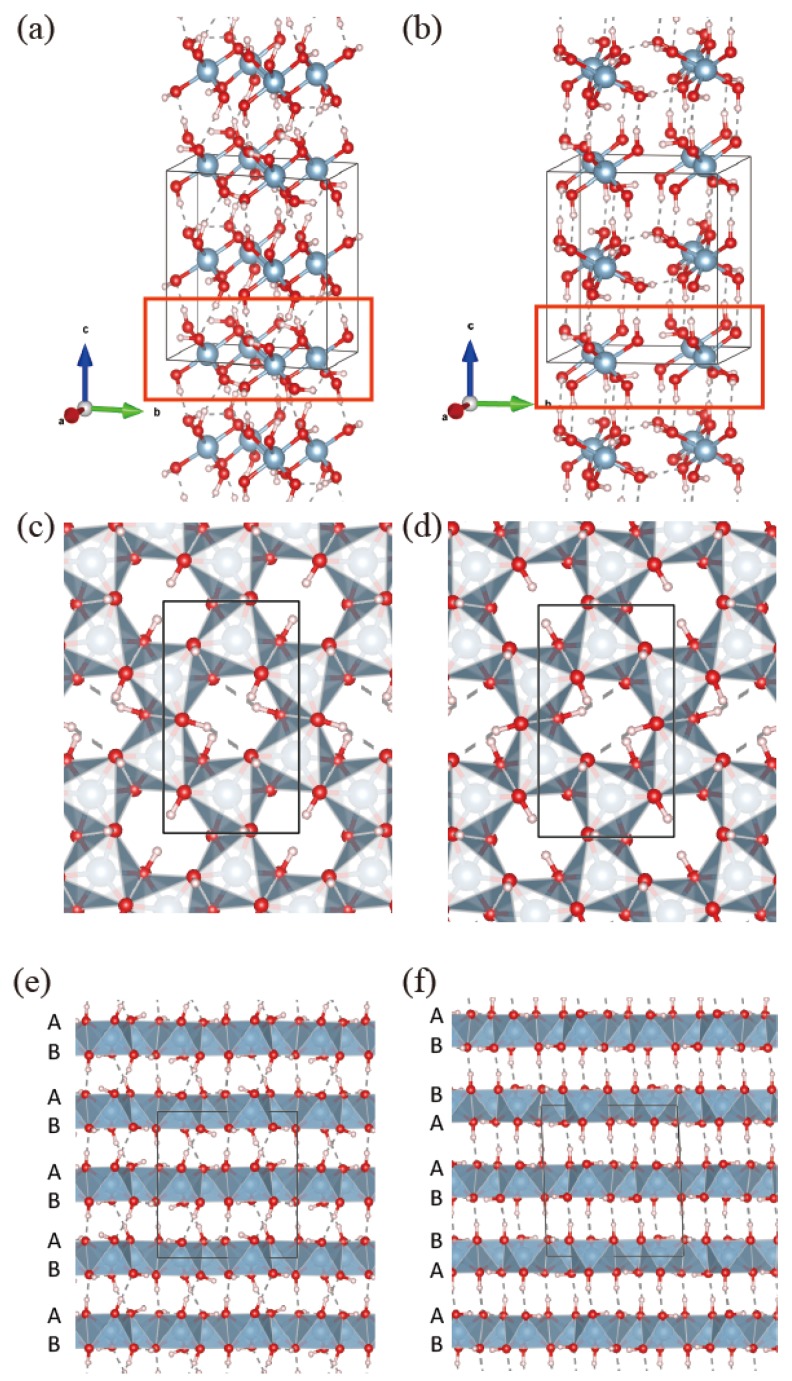
Crystal structure of (**a**) Bayerite and (**b**) Gibbsite. Two phases have an identical single layer as the building block. The same single layer is indicated in red rectangle in Bayerite and Gibbsite crystal structures; (**c**,**d**) Opposite faces of a single layer Al(OH)${}_{3}$(A and B sides, respectively). Octahedrons on two sides point in different directions; (**e**,**f**) Stacking sequence of Bayerite and Gibbsite. Bayerite is stacked by AB-AB sequence and Gibbsite is stacked by AB-BA sequence.

Aluminum hydroxide, Al(OH)${}_{3}$, is known to have a layered structure experimentally [18,19,20,21,22], and there are a few polytypes of Al(OH)${}_{3}$. However, there are not many reports on the first-principles calculations of the electronic band structures and their properties as layered materials. Among the polymorphs of aluminum hydroxide, Bayerite (sometimes designated as α-Al(OH)${}_{3}$) and Gibbsite (sometimes designated as γ-Al(OH)${}_{3}$) occur most frequently and we will focus on these two structures. Figure 1 shows the crystal structures of Bayerite and Gibbsite. It is experimentally known that both phases have a monoclinic unit cell. However, the values of β, the angle between lattice vector *a* and *c* of the unit cell are close to 90${}^{\circ }$ (experimental values are 90.27${}^{\circ }$ for Bayerite [27] and 94.57${}^{\circ }$ for Gibbsite [28], respectively). As presented in Table 1, values of β calculated via energy minimization for Bayerite and Gibbsite deviate from experiment by a small amount (0.2 and 2.4%, respectively). Bayerite and Gibbsite have an identical single layer unit indicated by a red box in Figure 1a,b as a building block for the bulk structure. In a single layer Al(OH)${}_{3}$, as shown in the top and bottom views in Figure 1c,d, Al atoms form honeycomb networks with a cavity at the center of the hexagon. Each Al atom has 6 nearest neighbor oxygen atoms comprising an octahedron. Each octahedron shares an edge with an adjacent octahedron. Hydrogen atoms form hydroxyl groups (OH) with oxygen atoms and may be located along either in-plane or out-of-plane directions. In-plane OH bonds give rise to distortions of the Al hexagon by adjusting the positions of oxygen and hydrogen atoms to minimize the total energy of the system. The OH bonds in the out-of-plane direction contribute to the interlayer hydrogen bonding between a hydrogen atom in OH and an oxygen atom in the next layer when layers are stacked. It is to be noticed that, as shown in Figure 1c,d, upper and lower sides of a single layer are distinct in structure. There are three oxygen atoms on each side of a single layer per oxygen octahedron and the geometry of oxygen atoms on the upper side (A) is different from that on the lower side (B). Note that the directions of the white triangles containing a grey aluminum atom in Figure 1c,d are opposite to each other. Therefore, it is possible to make distinctive phases with different stacking sequences, even though the building block of the bulk material is identical. Bayerite has an AB-AB stacking sequence and Gibbsite has a AB-BA sequence, where BA means an overturned structure of AB. Due to different stacking, two phases have a different geometry of interlayer hydrogen bonds and in turn all the lattice parameters are affected through the energy minimization. Parameters of the theoretically optimized structure for Bayerite, Gibbsite and an isolated single layer are presented in comparison with experiment in Table 1. Bayerite and Gibbsite have very similar in-plane lattice constants. A single layer Al(OH)${}_{3}$ exhibits slightly larger in-plane lattice constants (*a* and *b*) than the bulk case. When the same sides of the layer face each other (A-A or B-B) as in Gibbsite, interlayer hydrogen bonds are almost perpendicular to the plane as shown in Figure 1f. On the other hand, hydrogen bonds are supposed to be tilted between layers in Bayerite when two different sides face each other (A-B) as shown in Figure 1e and the interlayer distance (c/2) is accordingly decreased in Bayerite compared to Gibbsite. Note that the error in the theoretical calculation with respect to experiments for the lattice parameter *c* (3.2 and 2.2% for Bayerite and Gibbsite, respectively) is appreciably larger than the case of *a* and *b* (where the error is always within 1%). The interlayer interaction determines the *c* value for layered materials and it is difficult to describe the relatively weak interlayer interaction accurately. In our case, hydrogen bonding and the vdW interaction coexist and the error here (2–3%) seems a typical value which is rather difficult to improve further (at least, on the level of widely-used computational methods such as DFT-D2 employed here).

From DFT total energy calculations, we obtain the cohesive energy of an isolated layer of 2.60 eV/Å${}^{2}$ (relative to all isolated atoms). The exfoliation energies (to peel off a single layer from the surface of bulk Al(OH)${}_{3}$) are 43.3 and 45.3 meV/Å${}^{2}$ for Bayerite and Gibbsite, respectively. Hydrogen-bonded Bayerite and Gibbsite have a larger exfoliation energy than the cases bound mainly by the vdW interaction, 22 meV/Å${}^{2}$ for graphite and 28 meV/Å${}^{2}$ for h-BN using the same calculation scheme [29].

### 3.2. Electronic Band Structure of Gibbsite, Bayerite and Single Layer

**Figure 2:**
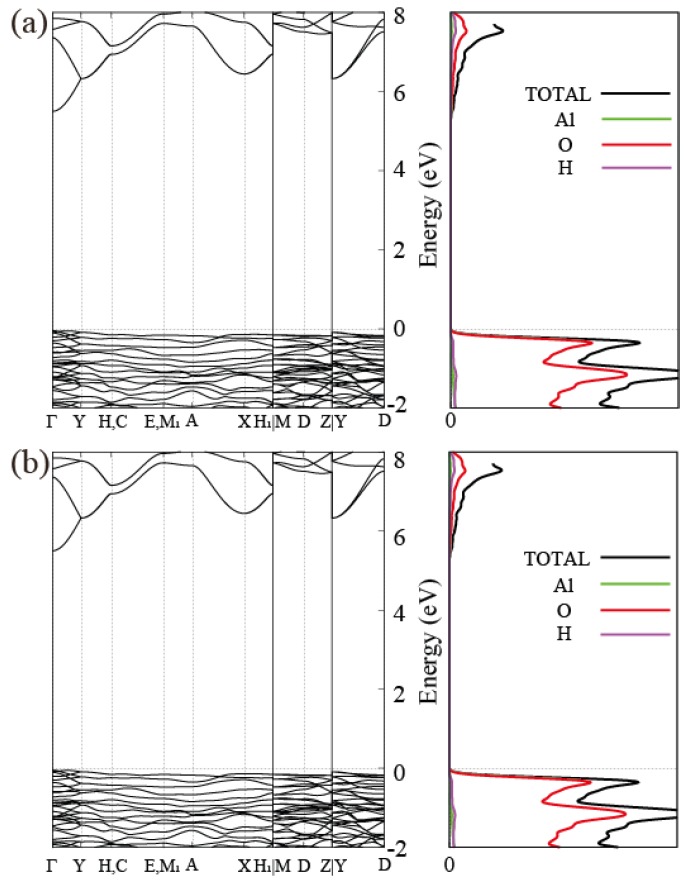
Calculated band structures and their projected density of states (PDOS) of (**a**) Bayerite and (**b**) Gibbsite. Bayerite is a direct gap insulator whereas Gibbsite is an indirect gap insulator. In both materials, valence bands are mostly composed of oxygen p-orbitals and conduction bands contain oxygen s and p, aluminum s, and hydrogen s, and p-orbitals.

**Figure 3:**
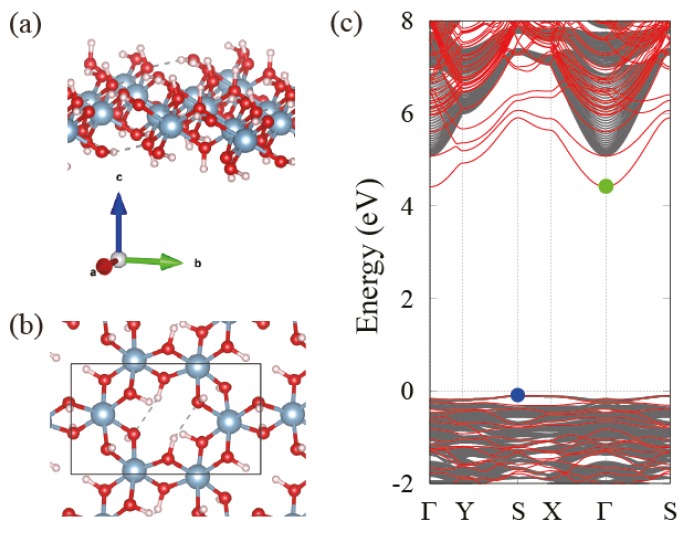
(**a**) Perspective view and (**b**) top view of the single layer Al(OH)${}_{3}$; (**c**) Band structure of single layer Al(OH)${}_{3}$ (red) juxtaposed with bulk spectrum (grey) projected onto the 2D Brillouin zone of a single layer.

**Figure 4:**
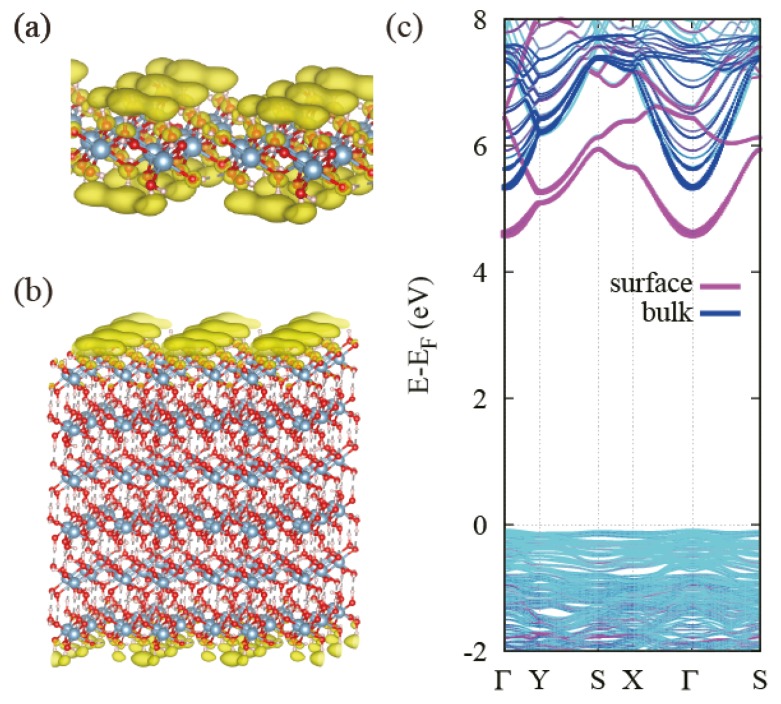
(**a**) Charge density of the conduction band edge state of single-layered Al(OH)${}_{3}$ and (**b**) that of multi-layered Al(OH)${}_{3}$; (**c**) Localization of the surface state of the slab structure in (**b**) based on the PDOS analysis. The surface region is defined as the top and bottom layers and the bulk region is the rest.

We obtain electronic band structures of Bayerite, Gibbsite and an isolated single layer using the optimized lattice parameters in Table 1. As shown in Figure 2, Bayerite and Gibbsite are both insulators with the calculated band gap of 5.54 and 5.27 eV, respectively. The projected density of states (PDOS) presented in Figure 2 indicates that valence bands are constituted mostly by oxygen p-orbitals and conduction bands are constituted mostly by oxygen s and p-orbitals with a little bit of Al and H atomic orbitals. Bayerite has a direct band gap at Γ-point. However, Gibbsite has its valence band maximum at E-point and the conduction band minimum at Γ, resulting in an indirect gap. We also present the electronic band structure of a single layer Al(OH)${}_{3}$ in Figure 3c. The band gap of the single layer is 4.46 eV, about 1 eV smaller than that of bulk Al(OH)${}_{3}$, and it is indirect. In order to figure out the reason for the gap decrease in the isolated layer, we project the 3D bulk band spectrum onto the 2D Brillouin Zone (BZ) of the single layer as indicated in grey in Figure 3c. By comparing two band structures, we identify a specific band in the single layer located below the bottom of the projected bulk conduction band continuum. We believe that this is a kind the surface state band created when a single layer is exfoliated from the bulk. To ensure that it is truly a surface band, we plot the real space charge density of the states of interest (the conduction band edge states) for the single-layer and multi-layer cases. In both cases, states at the conduction band edge are localized near the surface atoms in outermost layers and even outside of the layers (in vacuum) as shown in Figure 4a,b. We also project the Bloch wavefunctions of the multi-layered structure to atomic orbitals located at the surface (outermost layers) and bulk region, respectively. As shown in Figure 4c, Bloch states at the bottom of the conduction band in multi-layered structures are highly localized in the surface region. Surface localization and free-electron like dispersion of the band separated from the bulk band continuum are the characteristics of surface states and the existence of these states below the bulk conduction band minimum leads to the reduction of the band gap. We carry out a more detailed investigation of the behavior of the band gap in the next section.

### 3.3. Behavior of the Band Gap in Bulk and Few-Layer Al(OH)${}_{3}$

**Figure 5:**
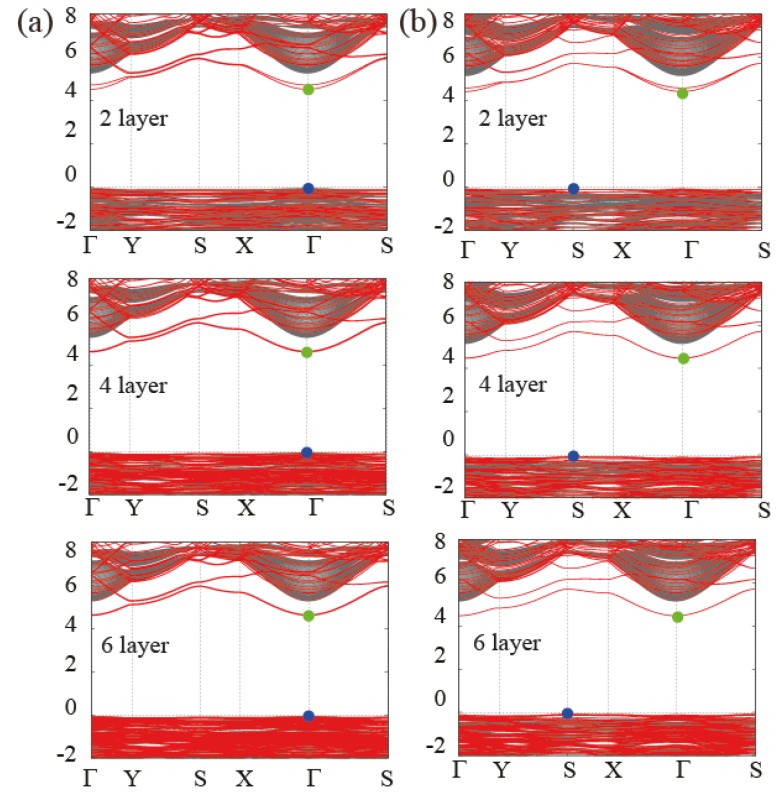
(**a**) Band structure of the bayerite-type 2, 4, and 6-layered slab geometry with 2D-projected bulk spectrum; (**b**) Band structure of the gibbsite-type 2, 4, and 6-layered slab geometry with 2D-projected bulk spectrum.

The band gap is one of the most important quantities in studying optical properties of materials. Many researchers are interested in tunability of the band gap in layered materials (see, for instance, Ref. [30,31,32]). As mentioned above, the band gap change from bulk to single layer is as much as 1.10 eV and 0.79 eV for Bayerite and Gibbsite, respectively. Single layer Al(OH)${}_{3}$ has an indirect band gap of 4.46 eV between the conduction band minimum at Γ and the valence band maximum at S. Bulk Bayerite has a direct gap at Γ, but bulk Gibbsite has a indirect gap between the conduction band minimum at Γ and the valence band maximum at E (which is projected to the S-point in 2D BZ). In order to clarify this issue more definitely, we additionally performed band structure calculations for multi-layered structures with an increasing number of layers (2, 4 and 6) with Bayerite and Gibbsite stacking sequences, respectively. The results are presented in Figure 5. In all finite-layered structures with the surface, we find the conduction band minimum state at Γ similar to the characteristic states found in the single-layer case. The behavior of the band gap is found to depend on the position of the valence band maximum with an increasing number of layers. Irrespective of the number of stacked layers, the valence band maximum of Gibbsite-type stacked multi-layers remains at S-point (onto which E-point of the bulk BZ is projected). In contrast, the valence band maximum of Bayerite-type multi-layers moves from S-point to Γ-point when the number of layers is 2 or above, making the structure a direct gap material.

If we examine the band structure of multi-layer Al(OH)${}_{3}$ closely, we can observe energy splitting of surface states due to the interaction between two surface states localized at opposite sides. When the number of layers increases, the interaction between surface states on opposite sides decreases and the energy splitting decreases, resulting in the increase of the band gap. For example, in the 6-layer case, the band gaps are 4.68 and 4.55 eV for Bayerite- and Gibbsite-type stacked multi-layers which is 0.21 and 0.10 eV larger than the band gap of the isolated layer, respectively, as shown in Figure 5a,b. It may provide a method to tune the band gap of Al(OH)${}_{3}$, although the allowed range of the band gap change is relatively small.

### 3.4. Alkali-Halide Intercalated Al(OH)${}_{3}$

**Figure 6:**
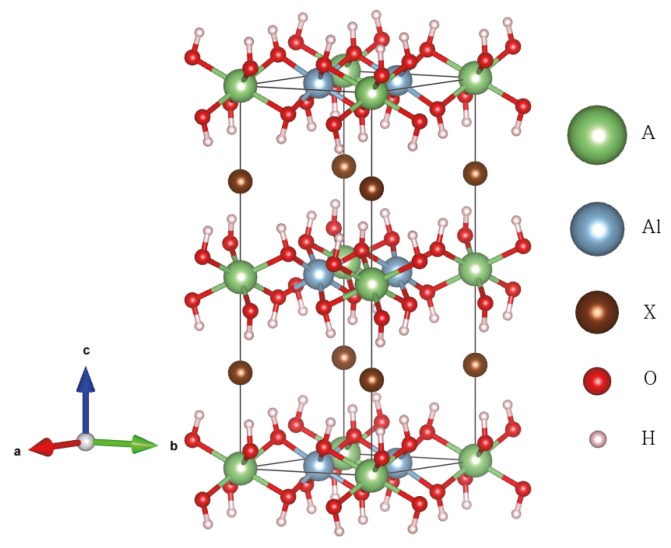
Crystal structure of alkali-halide intercalated Gibbsite (A[Al(OH)${}_{3}$]${}_{2}$X). An alkali metal atom is located at the center of the Al hexagon and a halogen atom is in the middle of two adjacent layers just above the alkali-metal atom.

Many researchers have investigated layered materials for applications, say, energy storage, gas adsorption, energy conversion, or support for catalysts. Layered materials usually have a higher surface to volume ratio than 3D bulk materials, provided that foreign molecules can penetrate into the interlayer region. Therefore, the interlayer distance would be a critically important factor and controlling the distance is essential to applications. Now, we consider the alkali-halide intercalation as a method to modify and tailor the interlayer distance of Al(OH)${}_{3}$, which in turn affects the electronic structure as well. Experimental results of alkali-halide intercalation in gibbsite are available in the literature [33,34,35]. Together with the intercalated alkali metal, the resulting material may be regarded a layered double hydroxide. The crystal structure of alkali-halide intercalated Al(OH)${}_{3}$ is shown in Figure 6. Alkali metal atoms are located on the cavity site at the center of the hexagon composed of six aluminum atoms, and halogen atoms are positioned in the middle of two adjacent layers just above the alkali-metal atoms. We study alkali-halide (AX) intercalation into the α and γ-phase Al(OH)${}_{3}$ using Li, Na, and K alkali (A) atoms and Cl, Br, and I halogen (X) atoms, respectively. We obtain relaxed structures of A[Al${}_{2}$(OH)${}_{6}$]X, i.e., fully doped alkali-halide intercalated Al(OH)${}_{3}$. As shown in Table 2, the magnitude of the binding energy of the intercalant decreases with increasing atomic radius of the intercalated atoms. The negative sign means that the intercalation is exothermic. Relaxed structures show that the atomic radius of intercalation atoms affects the lattice constant systematically. As the atomic size of the intercalated alkali metal (Li, Na and K) increases, the in-plane lattice constant *a* expands, as intuitively expected. In addition, as expected, the size of the halogen atoms (Cl, Br and I) is correlated positively with the interlayer distance (c/2) as evident in Table 2. There exists only limited experimental data available in the literature to our knowledge [34]. Starting from the Gibbsite structure (*a* = 5.08 and *c* = 9.74 Å [28]), they obtained *a* = 5.10 and *c* = 14.30 Å for Li[Al${}_{2}$(OH)${}_{6}$]Cl, and *a* = 5.10 and *c* = 14.95 Å for Li[Al${}_{2}$(OH)${}_{6}$]Br, respectively. In this limited example, our calculation agrees with the experimental trends. On the other hand, when the atomic radius of the alkali-metal increases, our calculation indicates that the lattice constant *c* decreases (no experimental data available for this), which appears somewhat counter-intuitive. It turns out that it is the result of the optimization of the whole unit cell, particularly related to the available volume of the space for halide atoms. Because the lattice constant *a* increases significantly by an increased atomic size of alkali metal atoms (say, K compared to Li), there occurs a compressive force in the perpendicular direction (*c*-axis) to limit too much expansion of the unit cell volume. The total unit cell volume (V), of course, increases as the alkali-metal size increases from Li to Na to K. Another interesting observation is the symmetry enhancement by intercalation. The intralayer and interlayer hydrogen bonds in pristine Bayerite or Gibbsite distort the hexagonal alignment of Al atoms (and the oxygen atoms connected to them). Thus, the geometry of them deviates from a flat regular hexagon and the crystal structure is monoclinic. As the intercalated atoms break intralayer and interlayer hydrogen bonds, the distortion of hexagonal alignment of Al atoms disappear. As a result, the hexagonal symmetry of the layer is achieved and the crystal structure symmetry is enhanced from monoclinic to hexagonal as shown in Figure 6, coinciding with the results of the above experiments for LiCl and LiBr intercalation [34].

### 3.5. Electronic Band Structure of Alkali-Halide Intercalated Al(OH)${}_{3}$

**Figure 7:**
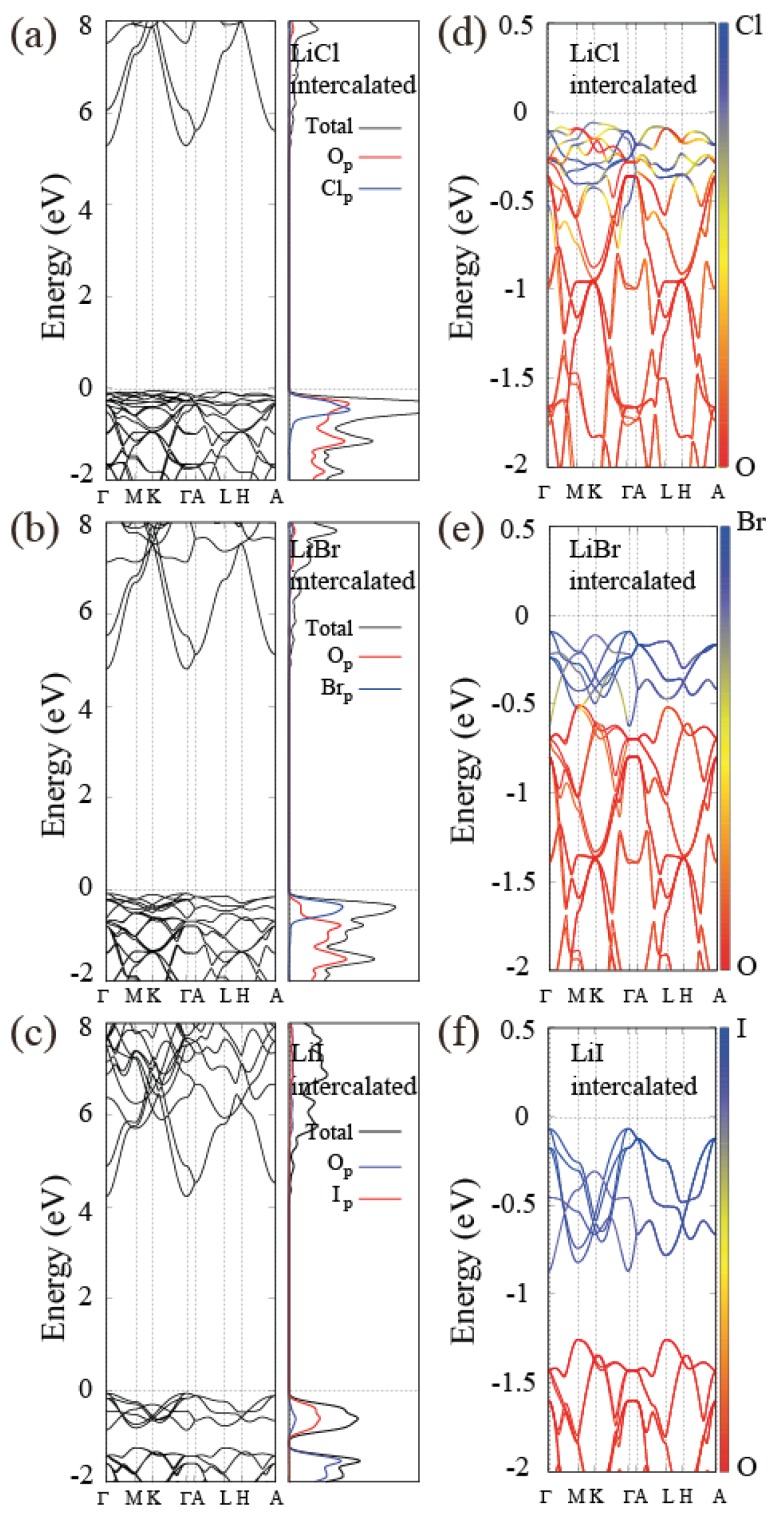
Electronic band structure and PDOS for (**a**) Li[Al(OH)${}_{3}$]${}_{2}$Cl; (**b**) Li[Al(OH)${}_{3}$]${}_{2}$Br; (**c**) Li[Al(OH)${}_{3}$]${}_{2}$I. The orange and purple lines indicate PDOS of oxygen p and halogen p orbitals, respectively. Decomposition of the orbital character into oxygen p and halide p orbitals in valence bands is represented by the color map using orange (O) and purple (Li, Br, and I) for (**d**) Li[Al(OH)${}_{3}$]${}_{2}$Cl; (**e**) Li[Al(OH)${}_{3}$]${}_{2}$Br; and (**f**) Li[Al(OH)${}_{3}$]${}_{2}$I. The hybridization between oxygen p and halogen decreases as the size of halogen atoms increases due to enhanced on-site energy of halogen p orbitals.

Intercalation affects not only the crystal structure of layered materials but also corresponding electronic band structures. We calculate band structures of all the materials listed in Table 2. The band gap of alkali-halide intercalated Al(OH)${}_{3}$ changes depending on the kinds of intercalated atoms, and the band gap of A[Al(OH)${}_{3}$]${}_{2}$X evolves to a smaller size when larger atoms are intercalated (for both alkali-metal and halogen atom cases). In detail, alkali-metal atoms contribute to conduction bands and the lower part of the valence bands as seen in the PDOS analysis in Figure 7. However, a significant contribution from halogen atoms appears at conduction and valence bands close to the band gap. There exists strongly hybridized Bloch states at the bottom of the conduction band region, showing a sizable halogen p-orbital portion with some mixture of oxygen, aluminum, and hydrogen atomic orbitals. In contrast, valence bands are mainly composed of oxygen p orbitals and halogen p orbitals. Figure 7a–c show the band structures and the PDOS with the LiCl, LiBr, and LiI intercalation and Figure 7d–f show the projection of Bloch wavefunctions to oxygen p and halogen p orbitals. The hybridization between oxygen p and halogen p orbital decreases as the on-site energy of the halogen p orbital increases from the Cl 3p to Br 4p to I 5p orbitals with respect to fixed on-site energy of oxygen p-orbitals and the character of the states near the top of the valence band is dominated by halogen p-orbitals. In the case of LiI intercalation, we notice in Figure 7 that the oxygen p bands and iodine p bands are completely split, which is largely responsible for the smaller band gap of iodine intercalated Al(OH)${}_{3}$ compared to other cases.

## 4. Conclusions

We have investigated aluminum hydroxide from the view point of quasi-2D layered materials. Although Bayerite and Gibbsite are composed of the same building block (an identical single layer Al(OH)${}_{3}$), different stacking sequences lead to somewhat different hydrogen bonding configurations. There exists hydrogen bonding between constituent layers of Al(OH)${}_{3}$ which gives rise to a stronger interlayer binding energy than that of typical vdW-interaction-mediated layered materials. From the single-layered and multi-layered structure calculations, we find that exfoliation (or creation of a new surface) induces surface localized states below the bottom of the conduction band, which reduce the band gap. We suggest alkali-halide intercalation for the purpose of tailoring the electronic band structure as well as the crystal structure. We have examined here some interesting properties of Al(OH)${}_{3}$ as a prototype hydrogen-bonded layered material and expect that there will be further interest and investigation in layered materials with hydrogen bonding as a major interlayer binding mechanism.

## Figures and Tables

**Table 1:** Unit cell information for the bulk and isolated single layer of Al(OH)${}_{3}$ obtained from first-principles calculations. Experimental data are presented wherever available. V is the unit cell volume.

Cal./Exp.	Bayerite	Gibbsite	Single Layer
*a*(Å)	5.03/5.06 ${}^{1}$	5.03/5.08 ${}^{2}$	5.08
*b*(Å)	8.66/8.67 ${}^{1}$	8.65/8.68 ${}^{2}$	8.68
*c*(Å)	9.12/9.42 ${}^{1}$	9.53/9.74 ${}^{2}$	-
β(${}^{\circ }$)	90.45/90.27 ${}^{1}$	92.34/94.54 ${}^{2}$	-
V(Å${}^{3}$)	397.27/413.25 ${}^{1}$	414.50/427.98 ${}^{2}$	-

${}^{1}$ Ref. [27], ${}^{2}$ Ref. [28].

**Table 2:** Unit cell information and the intercalation energy per alkali-halide unit (ΔBE) and the band gap for Bayerite/Gibbsite.

Alkali-Halide Intercalated Al(OH)${}_{3}$			
Bayerite/Gibbsite	Li[Al${}_{2}$(OH)${}_{6}$]Cl	Li[Al${}_{2}$(OH)${}_{6}$]Br	Li[Al${}_{2}$(OH)${}_{6}$]I
*a*(Å)	5.12/5.12	5.12/5.13	5.12/5.12
*c*(Å)	13.90/14.21	14.58/14.62	15.02/15.52
V(Å${}^{3}$)	315.72/322.03	331.05/332.75	341.44/351.93
ΔBE (eV)	−8.80/−8.64	−8.39/−8.23	−7.81/−7.80
Band gap (eV)	5.37/5.35	4.90/4.90	4.51/4.30
Bayerite/Gibbsite	Na[Al${}_{2}$(OH)${}_{6}$]Cl	Na[Al${}_{2}$(OH)${}_{6}$]Br	Na[Al${}_{2}$(OH)${}_{6}$]I
*a*(Å)	5.28/5.28	5.28/5.28	5.28/5.28
*c*(Å)	13.46/13.64	13.94/14.34	14.69/15.17
V(Å${}^{3}$)	324.71/329.84	336.86/346.67	355.22/366.03
ΔBE (eV)	−7.13/−6.95	−6.62/−6.50	−6.06/−6.00
Band gap (eV)	5.12/5.15	4.80/4.84	4.45/4.32
Bayerite/Gibbsite	K[Al${}_{2}$(OH)${}_{6}$]Cl	K[Al${}_{2}$(OH)${}_{6}$]Br	K[Al${}_{2}$(OH)${}_{6}$]I
*a*(Å)	5.48/5.48	5.47/5.48	5.47/5.48
*c*(Å)	12.90/13.04	13.58/13.93	14.35/14.75
V(Å${}^{3}$)	335.00/339.15	351.89/362.48	372.15/384.08
ΔBE (eV)	−4.22/−4.01	−3.74/−3.59	−3.21/−3.14
Band gap (eV)	4.98/4.90	4.63/4.62	4.25/4.20
